# Energy drink use is associated with alcohol and substance use in eighth, tenth, and twelfth graders

**DOI:** 10.1016/j.pmedr.2016.06.019

**Published:** 2016-06-29

**Authors:** Kathryn Polak, Pamela Dillon, J. Randy Koch, Willis G. Miller, Leroy Thacker, Dace Svikis

**Affiliations:** a Department of Psychology, Virginia Commonwealth University, Richmond, VA, United States; b Center for Clinical and Translational Research, Virginia Commonwealth University, Richmond, VA, United States; c Center for the Study of Tobacco Products, Virginia Commonwealth University, Richmond, VA, United States; d School of Education, Virginia Commonwealth University, Richmond, VA, United States; e School of Nursing, Virginia Commonwealth University, Richmond, VA, United States; f Institute for Women's Health, Virginia Commonwealth University, Richmond, VA, United States

**Keywords:** Adolescent health, Caffeine, Energy drinks, Alcohol, Tobacco, Drugs

## Abstract

The increasing prevalence of energy drink (ED) use and its link with negative behaviors and adverse health outcomes has garnered much attention. Use of EDs combined with alcohol among college students has been of particular interest. It is unclear if these relationships develop in the context of college, or if similar associations exist in younger individuals. The present study examined associations between ED consumption patterns and other substance use in an adolescent, school-based sample. Participants were *N* = 3743 students attending 8th, 10th or 12th grade in a suburban central Virginia public school system who completed a prevention needs assessment survey in 2012. Chi-square analyses and logistic regressions were used to compare rates of alcohol, tobacco and other drug use across three ED use groups: moderate/heavy (12.6%), light (30.5%), and non-users (57%). Over 40% of the sample reported recent (past month) ED use, with males more likely to report moderate/heavy ED use than females (14.0% and 11.1%, respectively; *p* = 0.02). After adjusting for gender and grade, ED use group predicted lifetime alcohol, tobacco and other drug use (all *p* < 0.001). Moderate/heavy ED users were most likely and ED non-users were least likely to report using each of the 13 substances in the survey, with light ED users intermediate to the other two groups. Moderate/heavy ED users were consistently most likely to report licit and illicit substance use. Additional research is needed to better understand which adolescents are at greatest risk for adverse health behaviors associated with ED use.

## Highlights

• Over 40% of the adolescent sample reported past month energy (ED) use.
• Males were more likely to report moderate/heavy ED use than females.
• Moderate/heavy ED users were most likely to report lifetime substance use, followed by light ED users and finally non-users.

## 1 Introduction

As the popularity of caffeinated energy drink (ED) use continues to increase, so do concerns about possible adverse effects. Many EDs contain substantive amounts of caffeine and heavy caffeine consumption can lead to such adverse effects as agitation/jitteriness, insomnia, tachycardia, and muscle tremors (Reissig et al., 2009, Arria and O'Brien, 2011). Monitoring has proven difficult, however, as many ED products are marketed as conventional foods, which do not require reports of serious adverse events to the FDA.

EDs are particularly popular among college students, with about two-thirds (65.5%) reporting ED use (e.g., Arria et al., 2011). ED use has been linked to heavier drinking, use of illicit and prescription drugs and other risk behaviors (e.g., Arria et al., 2011). Furthermore, college students who consume EDs combined with alcohol are more likely to use marijuana, meet criteria for alcohol dependence, and engage in other hazardous behaviors (Arria et al., 2010, Arria et al., 2011). ED marketing efforts often focus on young people, and ED consumption has increased among adolescents (Harris and Munsell, 2015, Pennington et al., 2010), with 30 to 50% of teens reporting ED use (Seifert et al., 2011). With increased use has come increased problems, and adolescents make up a significant portion of those negatively impacted by heavy caffeine use. In 2011, nearly 1500 energy-drink-related emergency department visits by individuals aged 12 to 17 were reported (SAMHSA, 2013). In Canada, ED use in adolescents has been linked to alcohol, tobacco and other drug use, as well as sensation-seeking and recent traumatic brain injury (TBI) (Hamilton et al., 2013, Ilie et al., 2015).

Despite increasing prevalence of ED use among adolescents, research has been sparse, with ED use often defined in broad terms (any use, lifetime) that may lack the specificity needed to better understand correlations between ED, other substance use and adverse consequences (Striley and Khan, 2014). Adolescent vulnerability for drug use and problems makes them an important target for substance abuse prevention efforts. The present study examined associations between ED and other substance use in a sample of 8th, 10th and 12th graders. Specifically, we compared rates of alcohol, tobacco and other drug use across 3 ED consumption groups. We hypothesized that rates of alcohol, tobacco, and other drug use would be highest among the moderate/heavy ED users, followed by light ED users, and finally non-ED users.

## 2 Method

### 2.1 Participants

Participants were *N* = 3743 students attending 8th, 10th, or 12th grade in one suburban, central Virginia public school system participating in the 2012 Prevention Needs Assessment Survey (representing over three-fourths (78%) of students in the school district).

### 2.2 Procedure

Parents of eligible participants were informed about the dates and purpose of the survey via an email. Assurances were given that students and individual schools would not be identified. A waiver of documentation of parental approval was used with passive consent procedures to obtain parental permission for students to complete the survey. On the day of administration, the survey was sent out to randomly selected 8th, 10th, and 12th grade classrooms and all students in attendance whose parents did not opt them out of participation were invited to complete the 45-minute, anonymous, paper-and-pencil survey during the school day. Prior to survey administration, students were reminded that the survey was anonymous and participation was voluntary.

Survey content focused on school experiences, peer interactions, family influences, community environment, risky behaviors, and substance use, including ED, alcohol, tobacco, and other drugs. The survey was developed by an organization dedicated to community-based prevention activities. Prior to administration, survey content was reviewed and approved by the school board, as well as the central Virginia school system and Institutional Review Board.

### 2.3 Measures

Measures included demographic, ED, alcohol, tobacco, and other drug use variables from the anonymous survey. The ED use item, “On how many occasions (if any) have you had an energy drink (e.g., Monster, Red Bull) during the past 30 days?” (with response options: 0, 1–2, 3–5, 6–9, 10–19, 20–39, and 40 +) was used to categorize students into one of three past month ED use groups: non-users (0 times, *N* = 1693; 57%), light ED users (1–5 times, *N* = 905; 30.5%), and moderate/heavy ED users (6 or more times, *N* = 374; 12.6%). For all other substances (see Table 1), participants were labelled “users” if they reported using that particular substance 1 or more times (lifetime).

### 2.4 Data analysis

Demographic (grade and gender) and other substance use data were compared across the 3 ED use groups using chi-square analyses. Then, logistic regression was used to predict alcohol, tobacco, and other drug use across the three ED use groups. Repeated contrasts were performed for the ED use variable, comparing non-ED users to light ED users, moderate/heavy ED users to non-users, and light ED users to moderate/heavy users. All analyses were carried out using SPSS version 21. For all reported analyses, Bonferroni corrections were applied to reported *p*-values and confidence intervals to adjust for the multiple comparisons and maintain an overall 0.05 family-wise error rate. Items left blank or with multiple responses were excluded from analyses involving that specific item.

## 3 Results

### 3.1 Sample demographics

The sample included *N* = 1447 (38.7%) 8th graders, *N* = 1225 (32.7%) 10th graders, and *N* = 1071 (28.6%) 12th graders. Mean age was 15.4 years (*SD* = 1.7) and 48.3% were female. The sample was predominantly White (59%), followed by African American (33.2%), Hispanic (10.9%), Native American (5.8%), and Asian (5.3%).

### 3.2 ED use by grade and gender

#### 3.2.1 Any ED use (lifetime)

The percent of adolescents who consumed EDs at least once increased from 64.5% in 8th grade to 73.5% in 10th grade, and 77.4% in 12th grade (*p* < 0.01). There were no gender differences, with nearly three-fourths of both males and females reporting ED use (70.2% vs 71.4%, respectively; NS).

#### 3.2.2 Any ED use (past month)

The percent of adolescents who consumed EDs at least once in the past month did not differ by age/grade level (45.5% vs 40.5% vs 41.8%, respectively; *p* = 0.06). However, males were more likely to report past month use than females (45.0% vs 41%, respectively; *p* = 0.03).

#### 3.2.3 Moderate/heavy ED use (past month)

Moderate/heavy ED use rates also did not vary significantly between 8th, 10th, and 12th graders (13.2%, 11.3%, and 13%, respectively; NS). However, males were more likely to report moderate/heavy ED use than females (14.0% vs 11.1%; *p* = 0.02). Also, male 12th graders were more likely to report moderate/heavy ED use (18.3%) than male 10th and 8th graders (10.4% and 13.5%, respectively; *p* < 0.01). No such relationship was found for females (*p* = 0.07).

### 3.3 Other substance use across 3 ED use groups

Rates of alcohol, tobacco, and other drug use for the full sample and the 3 ED use groups are summarized in Table 1. Consistently, across all 13 drug types, chi-square analyses found statistically significant differences between the 3 ED use groups.

### 3.4 ED use, grade level, and gender as predictors of substance use

To determine if the ED use group effect remained significant after adjusting for gender and grade level, logistic regression was used, including repeated contrasts (see Table 2). Specifically, ED use group predicted lifetime substance use (all *p* < 0.001) with significance found in nearly all contrasts. Grade level was predictive of lifetime substance use for each class of drugs. In addition, gender predicted lifetime substance use in several cases. Females were more likely to have tried alcohol, cigarettes, inhalants, and sedatives, while males were more likely to have tried LSD.

## 4 Discussion

### 4.1 Principal findings

The present study is among the first to quantitatively correlate ED use with adverse health behaviors. When participants were divided into three groups based on frequency of ED use (moderate/heavy, light, and no use), not only did rates of other substance use vary across the 3 ED use groups, but the pattern found was consistent for each of the 13 substances surveyed. In each case, as predicted, moderate/heavy ED users were most likely to use the substance, followed by light ED users and finally ED non-users. Specific contrasts between moderate/heavy and light ED users found some ORs were modest (e.g., 1.67 for amphetamines and 1.89 for marijuana/inhalants), others were moderate (e.g., 2.21 for cigarettes and 2.65 for tranquilizers), and still others were substantive (e.g., 4.22 for methamphetamines and 5.0 for heroin). This consistent dose-response like pattern affirmed moderate/heavy ED using adolescents were more likely to use all licit and illicit drugs surveyed, and might benefit from future substance abuse prevention efforts.

Present study findings are consistent with the much larger literature on college students and relationships found between ED use and problem drinking/other drug use (e.g., Arria et al., 2011). They are also largely consistent with previous research on adolescent ED use and associated problems (e.g., Azagba et al., 2014, Terry-McElrath et al., 2014). The present study found a higher proportion of students reported ED use than that found in the Canadian study by Azagba et al. (2014) (42% and 20%, respectively). Differences in assessment methods may contribute to this discrepancy, with Azagba et al. (2014) focused on past year ED use and the present study examining recent (past month) ED consumption. Agreement between adolescent reports on recent (past month) as compared to past year (average month) ED use warrants further study.

With a cross-sectional design, the present study does not posit a causal relationship between heavy ED and other substance use. The observed associations could represent many things, such as a general propensity toward addiction across a range of substances (e.g., Heath et al., 1997). Given that adolescent ED use is associated with sensation seeking (e.g., Hamilton et al., 2013), such correlations could also reflect a common proclivity toward risk taking. Alternatively, the notion that EDs could serve as a gateway drug, increasing likelihood of other drug use also warrants further study (Gallimberti et al., 2015).

### 4.2 Limitations

The present study relied upon retrospective self-report data, which are subject to recall bias. Further, the survey contained no quantity of ED use information. This limited our definition of moderate/heavy ED use to frequency. Nonetheless, the decision to categorize moderate/heavy ED use as consumption 6 or more times in the past month, or approximately 1 to 2 times per week, was consistent with research on moderate/heavy use of other substances (e.g., Miller, 2008). Another limitation was that other substance use was defined by any use (lifetime), which did not allow us to examine more substantive use or problematic use. Given the age groups in our sample, however, this appeared to be an appropriate starting point in the study of ED use subgroups. Lastly, our sample only included data from one suburban school district in Virginia, potentially limiting generalizability of study findings.

### 4.3 Future research

The current study presents benchmark data on the unique and elevated risks associated with moderate/heavy as compared to light and non-use of ED. While a link between ED use and risky behavior among youth has been previously identified (e.g., Meredith et al., 2015, Miller, 2008), the mechanisms governing these relationships remain unknown. Additional research is needed to better understand which adolescents are at greatest risk for adverse health behaviors associated with ED use. This information, if used to guide future prevention and intervention efforts, could have positive public health implications. Finally, quantity measures should be developed to more precisely define caffeine consumption and support more accurate and reliable research assessments.

## Conflict of interest statement

The authors declare that there are no conflicts of interest.

## Transparency document

Transparency document Image 1

## Figures and Tables

**Table 1:** Alcohol, tobacco, and other drug use by ED use group in one Central Virginia public school system in 2012.

Other substance use (Lifetime)	Overall rate (%)	Moderate/heavy ED use (6 + times) (%)	Light ED use (1–5 times) (%)	Non-ED Use (%)	χ^2^ Value (adjusted *p*-value)^a^
Alcohol	43.6%	63.0%	55.9%	32.9%	159.17 (< 0.001)
Cigarettes	28.1%	52.1%	34.0%	19.2%	186.92 (< 0.001)
Marijuana	28.7%	48.4%	34.6%	20.0%	148.45 (< 0.001)
LSD	5.1%	14.3%	5.3%	3.0%	78.04 (< 0.001)
Cocaine	2.7%	9.3%	3.1%	1.1%	78.01 (< 0.001)
Inhalants	11.5%	23.6%	14.5%	7.3%	89.97 (< 0.001)
Methamphetamine	1.4%	5.5%	1.3%	0.5%	56.18 (< 0.001)
Amphetamines	10.0%	20.1%	13.4%	5.7%	87.84 (< 0.001)
Sedatives	8.1%	21.7%	8.0%	5.0%	114.38 (< 0.001)
Tranquilizers	4.9%	13.5%	5.7%	2.5%	80.02 (< 0.001)
Prescription Narcotics	7.8%	17.3%	10.2%	4.3%	81.99 (< 0.001)
Heroin	1.1%	5.0%	1.0%	0.4%	53.83 (< 0.001)
Ecstasy	3.5%	8.5%	5.4%	1.8%	47.60 (< 0.001)

a Bonferroni correction applied.

**Table 2:** Main effects and odds ratios (with corresponding confidence intervals) comparing ED use groups in one central Virginia public school system in 2012.

	Main Effects						OR (98.33% CI) comparing ED use groups		
Substance	Gender		Grade		ED group	
χ^2^ value	Adjusted p-value^a^	χ^2^ value	Adjusted p-value^a^	χ^2^ value	Adjusted p-value^a^	Light vs none	Moderate/heavy vs none	Moderate/heavy vs light
Alcohol	32.78	< 0.0001^b^	134.47	< 0.0001	191.27	< 0.0001	3.12 (2.47, 3.95)	4.33 (3.08, 6.09)	1.39 (0.97, 1.99)^d^
Cigarettes	5.91	0.1956	159.69	< 0.0001	196.31	< 0.0001	2.43 (1.93, 3.08)	5.42 (3.99, 7.38)	2.23 (1.62, 3.06)
Marijuana	0.66	1.0000	296.30	< 0.0001	167.76	< 0.0001	2.51 (1.98, 3.20)	4.76 (3.46, 6.55)	1.89 (1.36, 2.63)
LSD	10.89	0.0125^c^	67.46	< 0.0001	65.01	< 0.0001	1.86 (1.12, 3.06)	5.55 (3.33, 9.25)	2.99 (1.77, 5.05)
Cocaine	1.90	1.0000	27.94	< 0.0001	58.60	< 0.0001	3.12 (1.50, 6.49)	9.81 (4.76, 20.19)	3.15 (1.66, 5.97)
Inhalants	14.61	0.0017^b^	22.87	< 0.0001	84.05	< 0.0001	2.15 (1.56, 2.97)	4.06 (2.79, 5.92)	1.89 (1.30, 2.76)
Methamphetamine	0.29	1.0000	7.16	0.0970	38.95	< 0.0001	2.92 (0.97, 8.77)^d^	12.40 (4.48, 34.29)	4.24 (1.74, 10.35)
Amphetamines	1.65	1.0000	96.17	< 0.0001	89.87	< 0.0001	2.80 (1.97, 3.98)	4.69 (3.09, 7.11)	1.67 (1.12, 2.51)
Sedatives	14.20	0.0021^b^	16.50	0.0006	106.60	< 0.0001	1.75 (1.17, 2.62)	5.88 (3.88, 8.89)	3.35 (2.18, 5.15)
Tranquilizers	0.00	1.0000	62.70	< 0.0001	71.29	< 0.0001	2.53 (1.51, 4.24)	6.73 (3.92, 11.54)	2.66 (1.58, 4.46)
Prescription narcotics	0.41	1.0000	114.95	< 0.0001	82.06	< 0.0001	2.82 (1.88, 4.21)	5.41 (3.41, 8.57)	1.92 (1.23, 3.00)
Heroin	0.94	1.0000	4.41	0.4644	35.92	< 0.0001	2.47 (0.74, 8.30)^d^	12.35 (4.19, 36.38)	5.00 (1.85, 13.48)
Ecstasy	1.24	1.0000	64.33	< 0.0001	43.23	< 0.0001	3.39 (1.91, 6.02)	5.41 (2.83, 10.35)	1.60 (0.88, 2.89)^d^

a Bonferroni correction applied.

b Significantly more females than males.

c Significantly more males than females.

d The confidence interval contains the value 1, which suggests there is no difference between the two ED use groups.

## References

[bb0005] Arria A.M., O'Brien M.C. (2011). The “high” risk of energy drinks. JAMA.

[bb0010] Arria A.M. (2010). Increased alcohol consumption, nonmedical prescription drug use, and illicit drug use are associated with energy drink consumption among college students. J. Addict. Med..

[bb0015] Arria A.M. (2011). Energy drink consumption and increased risk for alcohol dependence. Alcohol. Clin. Exp. Res..

[bb0020] Azagba S., Langille D., Asbridge M. (2014). An emerging adolescent health risk: caffeinated energy drink consumption patterns among high school students. Prev. Med..

[bb0025] Gallimberti L. (2015). Prevalence of substance use and abuse in late childhood and early adolescence: what are the implications?. Prev. Med. Rep..

[bb0030] Hamilton H.A., Boak A., Ilie G., Mann R.E. (2013). Energy drink consumption and associations with demographic characteristics, drug use and injury among adolescents. Can. J. Public Health.

[bb0035] Harris J.L., Munsell C.R. (2015). Energy drinks and adolescents: what's the harm?. Nutr. Rev..

[bb0040] Heath A.C. (1997). Genetic and environmental contributions to alcohol dependence risk in a national twin sample: consistency of findings in women and men. Psychol. Med..

[bb0045] Ilie G. (2015). Energy drinks, alcohol, sports and traumatic brain injuries among adolescents. PLoS One.

[bb0050] Meredith S.E., Sweeney M.M., Johnson P.S., Johnson M.W., Griffiths R.R. (2015). Weekly energy drink use is positively associated with delay discounting and risk behavior in a nationwide sample of young adults. J. Caffeine Res..

[bb0055] Miller K.E. (2008). Energy drinks, race, and problem behaviors among college students. J. Adolesc. Health.

[bb0060] Pennington N., Johnson M., Delaney E., Blankenship M.B. (2010). Energy drinks: a new health hazard for adolescents. J. Sch. Nurs..

[bb0065] Reissig C.J., Strain E.C., Griffiths R.R. (2009). Caffeinated energy drinks-a growing problem. Drug Alcohol Depend..

[bb0070] Seifert S.M., Schaechter J.L., Hershorin E.R., Lipshultz S.E. (2011). Health effects of energy drinks on children, adolescents, and young adults. Pediatrics.

[bb0075] Striley C.W., Khan S.R. (2014). Review of the energy drink literature from 2013: findings continue to support most risk from mixing with alcohol. Curr. Opin. Psychiatry.

[bb0080] Substance Abuse and Mental Health Services Administration [SAMHSA] (2013). Update on Emergency Department Visits Involving Energy Drinks: A Continuing Public Health Concern. http://archive.samhsa.gov/data/2k13/DAWN126/sr126-energy-drinks-use.htm.

[bb0085] Terry-McElrath Y.M., O'Malley P.M., Johnston L.D. (2014). Energy drinks, soft drinks, and substance use among United States secondary school students. J. Addict. Med..

